# Variant in the *PLCG2* Gene May Cause a Phenotypic Overlap of APLAID/PLAID: Case Series and Literature Review

**DOI:** 10.3390/jcm11154369

**Published:** 2022-07-27

**Authors:** Tatjana Welzel, Lea Oefelein, Ursula Holzer, Amelie Müller, Benita Menden, Tobias B. Haack, Miriam Groβ, Jasmin B. Kuemmerle-Deschner

**Affiliations:** 1 Division of Pediatric Rheumatology and Autoinflammation Reference Center Tuebingen (arcT), Department of Pediatrics, University Hospital Tuebingen, 72076 Tuebingen, Germany; tatjana.welzel@ukbb.ch (T.W.); lea.oefelein@student.uni-tuebingen.de (L.O.); 2 Pediatric Pharmacology and Pharmacometrics, University Children’s Hospital Basel (UKBB), University of Basel, 4031 Basel, Switzerland; 3 Pediatric Hematology and Oncology, University Children’s Hospital Tuebingen, 72076 Tuebingen, Germany; ursula.holzer@med.uni-tuebingen.de; 4 Institute of Medical Genetics and Applied Genomics, University of Tuebingen, 72076 Tuebingen, Germany; amelie.mueller@med.uni-tuebingen.de (A.M.); benita.menden@uni-tuebingen.de (B.M.); tobias.haack@med.uni-tuebingen.de (T.B.H.); 5 Center for Rare Diseases, University of Tuebingen, 72076 Tuebingen, Germany; 6 Institute of Immunodeficiency, Center for Chronic Immunodeficiency (CCI), Medical Center-University of Freiburg, Faculty of Medicine, University of Freiburg, 79106 Freiburg, Germany; miriam.gross@uniklinik-freiburg.de

**Keywords:** autoinflammation, immunodeficiency, *PLCG2* gene variant, cold-induced urticaria, next generation sequencing

## Abstract

Background: Variants in the *phospholipase C gamma 2* (*PLCG2*) gene can cause *PLCG2*-associated antibody deficiency and immune dysregulation (PLAID)/autoinflammation and *PLCG2*-associated antibody deficiency and immune dysregulation (APLAID) syndrome. Linking the clinical phenotype with the genotype is relevant in making the final diagnosis. Methods: This is a single center case series of five related patients (4–44 years), with a history of autoinflammation and immune dysregulation. Clinical and laboratory characteristics were recorded and a literature review of APLAID/PLAID was performed. Results: All patients had recurrent fevers, conjunctivitis, lymphadenopathy, headaches, myalgia, abdominal pain, cold-induced urticaria and recurrent airway infections. Hearing loss was detected in two patients. Inflammatory parameters were slightly elevated during flares. Unswitched B-cells were decreased. Naïve IgD+CD27− B-cells and unswitched IgD+CD27+ B-cells were decreased; switched IgD-CD27+ B-cells were slightly increased. T-cell function was normal. Genetic testing revealed a heterozygous missense variant (c.77C>T, p.Thr26Met) in the *PLCG2* gene in all patients. Genotype and phenotype characteristics were similar to previously published PLAID (cold-induced urticaria) and APLAID (eye inflammation, musculoskeletal complaints, no circulating antibodies) patients. Furthermore, they displayed characteristics for both PLAID and APLAID (recurrent infections, abdominal pain/diarrhea) with normal T-cell function. Conclusion: The heterozygous missense *PLCG2* gene variant (c.77C>T, p.Thr26Met) might cause phenotypical overlap of PLAID and APLAID patterns.

## 1. Introduction

Autoinflammatory diseases (AID) belong to a constantly growing spectrum of rare entities caused by gene variants, leading to abnormal activation of the innate immune system. Clinical characteristics include recurrent fevers, inflammation of joints, eyes, skin and serous membranes, coupled with increased inflammatory markers. Immunodeficiency is caused by a pathologically reduced immune function involving the innate and/or the adaptive immune system. Monogenic syndromes with concomitant symptoms of autoinflammation and immunodeficiency have been reported in recent years and reports are expanding [1].

Mutations in the *phospholipase C gamma 2* (*PLCG2*) gene might be associated with a spectrum of diseases, ranging from allergy and immunodeficiency to autoimmunity and autoinflammation [2]. In 2012, Ombrello et al. described three families with variants in the *PLCG2* gene affecting the autoinhibitory C-terminal Src-homology 2 (cSH2) domain, which was later proposed as PLCG2-associated antibody deficiency and immune dysregulation (PLAID) [3]. PLAID is typically characterized by cold-induced urticaria and symptoms of immune dysregulation [3,4]. In addition, the autoinflammation and PLCG2-associated antibody deficiency and immune dysregulation (APLAID) syndrome is caused by p.Ser707Tyr variants of the *PLCG2* gene located in the cSH2 domain [5]. Clinical characteristics include systemic and organ-specific inflammation and symptoms of immunodeficiency [5]. Nowadays, the availability of next generation sequencing allows the detection of gene variants in previously undiagnosed patients with characteristics of an AID. However, making a diagnosis is challenging, as the correlation between clinical phenotype, genotype and pathogenicity status of a detected variant must be taken into account [6]. Making a diagnosis is even more challenging when phenotype and genotype characteristics of a specific variant have not been described yet.

Here, we report a single center case series of five related patients with a history for AID and symptoms of immunodeficiency. Based on clinical, laboratory and genetic findings of the index patients, a literature review was performed, to investigate if the detected *PLCG2* gene variant may cause the clinical symptoms. The findings suggest a phenotypical overlap between the PLAID and APLAID patterns in these patients.

## 2. Material and Methods

This is a single center case series of five related patients transferred to a tertiary hospital for advanced diagnostic work-up, due to suspected AID. Patients’ data were captured standardized in the designated institutional web-based Arthritis and Rheumatism Database and Information System (ARDIS). Individual written patient’s informed consent was obtained for genetic testing and for this publication. Approval of the Institutional Review Board Tuebingen was obtained (608/2021A).

### 2.1. Case Series

The patients’ characteristics include the following: patient 1 is a 44-year-old woman. She is the mother of patient 2, a boy of 14 years and patient 3, an 11-year-old boy. In addition, the mother had 4-year-old twins, patient 4 (a boy) and patient 5 (a girl). All patients were Caucasians. They presented with fever almost daily, recurrent episodes of conjunctivitis, lymphadenopathy, headaches, myalgia, abdominal pain and diarrhea. Furthermore, they suffered from cold-induced urticarial and erythematous/red rashes at the trunk and extremities. All patients had a history of mild to moderate upper airway infections every two to three weeks and recurrent sinusitis. So far, hospitalization or antibiotic treatment have not been necessary, due to infections. The symptoms of the mother appeared in early adulthood and showed increasing severity over time, whereas her children were affected since infancy. Otherwise, the family history was unremarkable and there was no consanguinity between parents.

Regarding diagnostic work-up, clinical and laboratory investigations, high-frequency pure tone audiometry (HF-PTA), and ophthalmology examination were performed. Genetic studies included exome sequencing of the four siblings, followed by combined analysis for variant polarization. Details are provided in the supplementary materials (S1: targeted exome sequencing). Carrier testing of the mother was carried out by Sanger sequencing; PCR conditions and primer sequences are provided upon request. Peripheral blood was analyzed by flow cytometry (FACS) for lymphocyte subsets. Peripheral blood mononuclear cells (PBMCs) were harvested from peripheral blood with Lysing solution (BD Biosciences, Europe) and stained for cell surface markers using fluorochrome-conjugated antibodies (Supplementary Material S2). Samples were acquired using a FACS Calibur cytometer, and data were analyzed with CellQuest software (BD Biosciences, Europe). To examine T-cell function, the expression of Interleukin (IL)-2, -4, -17/interferon (IFN) γ, and carboxyfluorescein succinimidyl ester (CFSE) proliferation of T-cells were measured (Supplementary Material S2).

### 2.2. Literature Review

The database Infevers (https://infevers.umai-montpellier.fr/web/, latest access date 1 May 2022) [7] was screened to identify gene variants with a similar phenotype (autoinflammatory and immunodeficiency symptoms). As patients previously described with *PLCG2* gene variants diagnosed as PLAID or APLAID showed similar clinical phenotypes, a literature review in Pubmed (https://pubmed.ncbi.nlm.nih.gov) was performed to identify additional reports of patients diagnosed as PLAID or APLAID by using the search items (i) APLAID, (ii) *PLCG2* associated antibody deficiency and immune dysregulation, (iii) PLAID syndrome, (iv) *PLCG2* gene variants, (v) *PLCG2* and autoinflammation, (vi) *PLCG2* and immunodeficiency in April 2022. Data were analyzed for common clinical and laboratory characteristics if (i) written in English, (ii) phenotype and genotype characteristics were presented and if (iii) the data source was a case report/case series or original article with available fulltext. Publications, which did not link the reported clinical phenotype and genotype with PLAID or APLAID, were excluded. Publications indicating the diagnosis FCAS3 (familial cold autoinflammatory syndrome 3) were counted in line with Omim (https://www.omim.org/entry/614468) as PLAID. Review articles were excluded, as well as reports with concomitant additional variants in other genes.

## 3. Results

### 3.1. Case Series

Regarding the patients’ characteristics, we recorded whether the patients’ history included symptoms or signs suggestive of AID and immunodeficiency (Table 1).

For diagnostic work-up, conjunctivitis was confirmed by an ophthalmologist. HF-PTA revealed mild high-frequency hearing loss in patient 1 and early signs of high-frequency hearing loss in patient 2 (Table 1). Skin biopsy (patient 1) displayed diffuse thickening of the reticular dermis layer, single neutrophil granulocytes and perivascular edema compatible with urticaria. Blistering autoimmune dermatosis was excluded. Inflammatory laboratory parameters were non-contributory between disease flares, whereas all patients had mild to moderate elevated serum amyloid A (SAA) during flares and increased serum calprotectin (S100A8/A9) (Table 1). Whole blood count, liver enzymes, urine analysis and kidney function were normal. Creatine kinase, natural killer cells and immunoglobulins were normal or slightly increased (Table 1). Immunopathologic testing showed nonspecific stimulation of antibodies, but no presence of antinuclear antibodies (ANA) (Table 1). Additional immunological work-up, including extractable nuclear antibodies (ENA), anti-neutrophilic cytoplasmatic antibodies (ANCA), double strain desoxyribonucleotid acid antibodies, myositis antibodies, rheumatoid factor and anti-critrulline peptide (anti-CCP) antibodies, in patient 1 were all normal (Table 1). Furthermore, T-cell proliferation, Th1-, Th2- and Th17-associated cytokine profiles were normal (Table 1). Malignancies, autoimmune diseases, chronic infections and common immunodeficiencies were excluded. Extended laboratory tests did not indicate hypogammaglobulinemia or B-cell deficiency and sufficient pneumococcal titers. In addition, other vaccine responses were mostly sufficient; but patient 2 had an insufficient response for the vaccination against diphtheria, patient 3 for the vaccination against hepatitis B and patient 4 had insufficient vaccine response after been vaccinated against diphtheria and tetanus (Table 1). Unswitched B-cells were decreased, indicating immune system hyperreactivity. Naïve IgD+CD27− B-cells and unswitched IgD+CD27+ B-cells were decreased and switched IgD-CD27+ B-cells were slightly increased in all patients. T-lymphocytes were only reduced in patient 1 and slightly elevated in patients 4 and 5 (Table 1). T-cell function was unremarkable. Exome sequencing revealed a heterozygous missense variant (c.77C>T, p.Thr26Met) in the *PLCG2* gene in all five patients (supplementary material S3). In addition, five other variants associated with human diseases (*PTCH2, POMGNT1, EPRS1, TREX1, HCLS)* were identified. However, by screening the database Infevers for the *PLCG2* variant only, similar clinical features to those observed in the family of the case series were previously observed.

### 3.2. Literature Review

**Figure 1:**
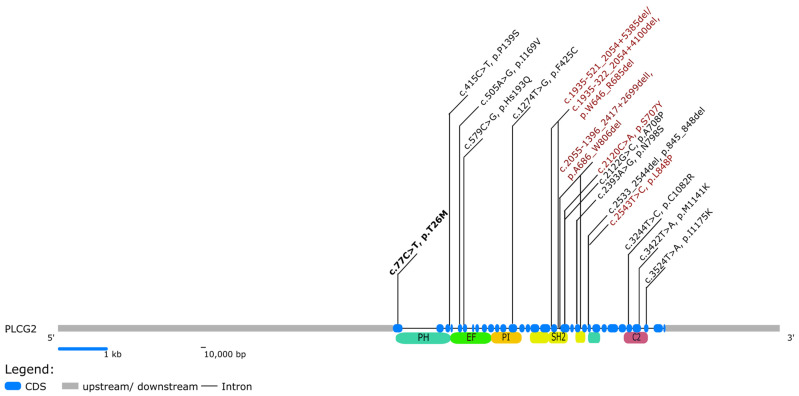
Structure of *PLCG2* with known protein domains and localization of variants associated with APLAID/PLAID. Variants listed in ClinVar as likely pathogenic or pathogenic are written in red and variants of uncertain significance in black. Abbreviations: CDS *coding sequence*, kb *kilobase*, bp *base pair*.

Of the thirty-one sequence variants in the *PLCG2* gene listed in Infevers [7], two pathogenic, one variant of unknown significance (VUS), one likely benign, and five so far not classified *PLCG2* gene variants displayed an APLAID phenotype and three not classified *PLCG2* gene variants presented a PLAID phenotype [3,5,8,9,10,11,12,13]. In addition, five other papers were identified in the PubMed search, which fulfilled the inclusion criteria [14,15,16,17,18]. Data fulfilling inclusion criteria were analyzed for genetic, clinical and immunology characteristics (Table 2 and Table 3). Several patients diagnosed as PLAID had evidence for cold-induced urticaria, increased susceptibility for recurrent infections, signs of autoimmunity and allergic diseases and immunologic findings (reduced immunoglobulins, low numbers of circulating class switched memory B-cells, reduced NK cells) [3,10,18]. Several patients diagnosed as APLAID had the following clinical and laboratory characteristics: cutaneous findings (rashes, erythema, granulomas, cutis laxa and vesiculo-pustular lesions), eye inflammation, recurrent infections, abdominal pain and inflammatory bowel disease, musculoskeletal complaints, immunologic findings (reduced/normal immunoglobulins, reduced circulating class-switched CD27+ memory B-lymphocytes and decreased/normal NK cells) [5,8,9,12,14,15,16,17,18]. Data were compared with patients’ characteristics from our case series, highlighting an overlap of characteristics previously reported for the PLAID and APLAID syndrome. In addition, four publications were identified during the search not to fulfill all the inclusion criteria. The authors did not link their observation with an APLAID/PLAID diagnosis due to differences in the observed phenotype, these publications were summarized in Table 4 [11,19,20,21] as they report phenotype and genotype characteristics, which might be associated with APLAID or PLAID. The gene structure of PLCG2 with the encoded protein domains and known disease-associated variants is provided in Figure 1.

### 3.3. Management

Based on clinical, laboratory and genetic findings, as well as the literature review, a phenotypical overlap between PLAID and APLAID disease patterns was suggested and the question of how the patients could be treated was raised. The available literature indicates that therapeutic management is challenging and that no generally effective therapeutic approach has been established so far. There is evidence, however, that in several patients with PLAID/APLAID, systemic corticosteroids might induce symptom improvement and partial disease control [5,8,9,14,18,22,23]. Furthermore, antihistamines, omalizumab, dapsone and hydroxychloroquine have been reported to improve particular skin features [4,8,9,14]. To prevent infectious diseases, immunoglobulin replacement and prophylactic antibiotic treatment have been reported [8,9,14,18]. With respect to inflammatory features, immunomodulatory treatment with interleukin (IL)-1 inhibition and tumor-necrosis-factor (TNF) inhibition have been reported. TNF inhibition was without effect in some patients, whereas others indicated partial response [5,8]. Similarly, results for IL-1 inhibition were heterogeneous; some patients experienced symptom improvement/partial response, whereas other patients did not [5,8,9]. In this case series, patient 1 was treated initially with short acting IL-1 inhibition (anakinra 100 mg s.c. every other day), due to her severe disease activity and the high frequency hearing loss. In patients 2, 4 and 5, treatment with long-acting IL-1 inhibition (canakinumab 2 mg/kg max. 100 mg every 8 weeks) was initiated. Patient 3 received colchicine (1 mg daily), as he had only mild to moderate disease activity with a physician global assessment (PGA) of 3 (PGA 0 = no disease activity, 10 = maximum disease activity) and colchicine has been reported to be effective in some other AID [24,25,26]. After canakinumab was started, patients 4 and 5 experienced significantly more infections (urinary tract infections, respiratory infections, ear infections) so that treatment was switched to colchicine (0.5 mg daily), as in the meantime satisfactory symptom control was achieved with colchicine in patient 3. Over time, treatment was adjusted to improve treatment response accordingly: patient 1 received anakinra 100 mg every other day and colchicine 1.5 mg daily, patient 2 received canakinumab 150 mg every 8 weeks and colchicine 1.5 mg daily, patient 3 had no need for treatment adjustment, patient 4 and 5 received colchicine 1 mg daily. This therapeutic approach did not lead to complete remission but in all patients, symptoms improved to mild disease activity (PGA ≤ 3).

## 4. Discussion

This is a case series of five related patients carrying the same heterozygous missense variant (c.77C>T, p.Thr26Met) in the PLCG2 gene, after exclusion of other possible causes. Based on clinical, laboratory and genetic findings, this variant might cause a phenotypical overlap between PLAID and APLAID disease patterns, when compared to previous reports. However, evidence of the pathogenicity of this variant seems controversial. In Varsome (https://varsome.com/variant/hg38/PLCG2%20Thr26Met, latest access 29 April 2022), the variant has been reported benign, whereas it is listed in Clinvar as a variant of uncertain significance (2×) and (likely) benign (1×) (https://www.ncbi.nlm.nih.gov/clinvar/variation/618829, latest access 29 April 2022). AlphaFold [27,28] predicts, for the missense changes, a location within a beta-sheet structure with very high confidence (pLDDT >90; Supplementary Material S4). Although a change of the aliphatic amino acid threonin to methionin might potentially affect the secondary protein structure, the in silico prediction is not highly damaging (CADD-score = 21, CADD version 1.5). In this case series, the heterozygous missense variant (c.77C>T, p.Thr26Met) in the PLCG2 gene seems to be causative for the phenotypical overlap between PLAID and APLAID. All five patients in this case series had clinical and laboratory similarities with previously described patients diagnosed as PLAID/APLAID. The patients in this case series showed PLAID characteristics, such as cold-induced urticaria [3,10] and APLAID characteristics, such as eye inflammation, musculoskeletal complaints and no circulating antibodies [5,8,9,12,14,15,16]. All patients had recurrent upper airway infections, abdominal pain, episodic diarrhea and normal T-cell function, as previously described in several PLAID and APLAID patients [3,5,8,9,12,14,15,16]. In addition, they had normal NK cells, similar to several APLAID patients [5,8,9,14]. However, in this case series, the immunoglobulins were normal and the patients had low unswitched B-cells, in contrast to the low class-switched B-cells described for PLAID/APLAID [3,5,8,9,12,14]. Furthermore, all patients displayed recurrent fever, fatigue and increased inflammatory parameters, all well-known autoinflammatory symptoms. Sensorineural hearing loss (SHL) was detected in patients 1 and 2. SHL was reported for one APLAID patient with a PLCG2 gene variant located in the pleckstrin homology (PH) domain [9]. Inflammation might be partially driven in APLAID patients by the NLRP3 inflammasome [29]. Nakanishi et al. demonstrated that the NLRP3 inflammasome can be activated in resident macrophage/monocyte-like cells in a mouse cochlea, resulting in IL-1β secretion with hearing loss [30]. Taken together, this newly identified PLCG2 gene variant might display a new phenotype with clinical and laboratory overlap of PLAID and APLAID symptoms.

This reported PLCG2 gene variant is located in the PH domain. The PLCG2 gene carries three Src homology (SH) and one split pleckstrin homology domain (spPH) between two subcatalytic subdomains (spPHn-SH2n-SH2c-SH3-spPHc). The spPH domain is involved in the GTPase Rac binding [31]. Typically, APLAID seems to be caused by the c.2120C>A (p.Ser707Tyr) variant of PLCG2 located within the cSH2 domain, whereas PLAID is caused by PLCG2 in-frame deletions located in the cSH2 domain [1]. Cells carrying the c.2120C>A (p.Ser707Tyr) sequence variant have only a small amount of enzymatic activity and they are not activated by cold exposure [1,5]. In contrast, PLAID-causing variants lead to consecutive activation when exposed to cold [3,22]. B-, NK- and mast cells bearing these PLAID variants are hypo-responsive to conventional stimulation at physiological temperatures, whereas cold exposure activates intracellular signaling and results in urticaria [3,22]. PLAID-associated PLCG2 activation by cold exposure seems to be dependent on the integrity and pliability of the spPH domain [32]. This assigns a regulatory role of the spPH domain in the mechanism of PLCG2 activation [32]. Additionally, single point variants in the murine PLCG2 gene within the spPH domain seem to lead to inflammation. This indicates that the location in the PH domain of this variant may explain some overlap of PLAID/APLAID characteristics.

This report has several limitations. This case series contains five patients with a rare disease (estimated prevalence <1/1,000,000; https://www.orpha.net). The majority of other published reports include one to three patients [5,8,9,10,12,14,15,16,17,18]. However, Ombrello et al. presented data of 27 patients from three families [3]. In addition, pathogenicity for the detected p.Thr26Met variant is so far inconclusive and has been reported as benign or as a variant of uncertain significance. In this case series, the variant seems to be causative for phenotypical APLAID/PLAID overlap in the five patients, strengthening the assumption that the variant might be of uncertain significance. For some IL-1-mediated AID, it has been reported that variants of uncertain significance can cause AID phenotypes in individuals, whereas other carriers do not show any symptoms [33,34,35]. In addition, the clinical presentation of patients with variants of uncertain significance may vary from those with (likely) pathogenic variants [33]. For the tumor-necrosis factor associated syndrome (TRAPS), the estimated prevalence is reported to be 1-9/1,000,000 (https://www.orpha.net, latest access 29 April 2022). However, for the R92Q (new nomenclature R121Q), higher frequencies (up to 5%) seem to be reported [36]. In all patients, an extensive diagnostic work-up was performed, data were captured standardized and a literature review was performed. Despite a comprehensive search strategy, there might be the risk of reporting bias and some data might have been missed by the defined search strings and inclusion/exclusion criteria. As previous reports have addressed different clinical and laboratory features, presentation of the characteristics was challenging. However, we discuss the main disease characteristics, therapeutic approaches, variability in disease symptoms and differences between APLAID/PLAID patients, although not all authors have referred to the same symptoms.

## 5. Conclusions

This case series describes a heterozygous missense variant (c.77C>T, p.Thr26Met) in the PLCG2 gene that may cause a phenotypical overlap of PLAID and APLAID, although the pathogenicity is currently unclear. A comprehensive literature review of PLAID/APLAID was performed to give an overview of the current knowledge about the clinical and laboratory characteristics. We conclude that further research and larger functional studies in PLCG2 gene variant-associated diseases are important to improve understanding and to facilitate the determination of a diagnosis in these rare diseases.

## Figures and Tables

**Table 1:** Clinical and laboratory characteristics of patients with the heterozygous missense *PLCG2* variant (c.77C>T, p.Thr26Met).

Clinical and Laboratory Characteristics	Pat 1 (f, 44 y.)		Pat 2 (m, 14 y.)		Pat 3 (m, 11 y.)		Pat 4 (m, 4 y.)		Pat 5 (f, 4 y.)	
**Clinical Symptoms**										
Recurrent fevers	✓		✓		✓		✓		✓	
Headache	✓		✗		✓		✗		✗	
High frequency hearing loss	✓		✓		✗		✗		✗	
Conjunctivitis	✓		✓		✓		✓		✓	
Painful recurrent lymph nodes	✓		✗		✗		✓		✓	
Episodes with abdominal pain and diarrhea	✓		✗		✓		✓		✗	
Cold-induced urticaria	✓		✗		✓		✓		✓	
Myalgia/ arthralgia	✓		✓		✓		✗		✗	
Recurrent upper airway infections, sinusitis	✗		✓		✓		✓		✓	
Recurrent swelling of palms and feet	✗		✗		✓		✓		✗	
Fatigue	✓		✗		✗		✓		✓	
**Symptom Onset**										
Symptom Onset	Early Adulthood		Infancy							
**Laboratory Parameters**	no flare	flare	no flare	flare	no flare	flare	no flare	flare	no flare	flare
CRP (mg/dL, reference max 0.5 mg/dL)	0.25	-	0.09	-	0.5	0.79	0.01	-	0.02	0.6
Soluble IL-2 R (U/mL, reference 158–613 U/mL)	172.0	-	266.0	-	644.0	-	560.0	-	636.0	-
CK (U/L, reference <170 U/L)	173	112	190	-	186	-	166	-	157	-
Calprotectin (S100 A8/A9) (µg/mL, reference <3 µg/mL)	4.8	-	1.8	5.65	3.6	-	1.6	-	2.1	-
Serum amyloid A (mg/L, reference <10 mg/L)	5	16.0	-	24	9	18	-	24	6	40
Lymphocytes (%)	16.2	-	35.5	-	36.3	39.3	49.4	-	54.4	28.6
Monocytes (%)	13.9	-	8.0	-	8.6	12.1	7.2	10.4	5.2	8.1
**Leucocytes**										
-Abs. neutros. (thousand/µL)	2.74 (2.1–77)		2.65 (2.0–6.6)		1.87 (1.8–6.6)		2.37 (1.8–7.4)		1.76 (1.8–6.8)	
-Abs. lymphos. (thousand/µL)	0.86 (1.2–3.5)		1.99 (1.1–3.4)		1.94 (1.1–3.4)		3.26 (1.3–4.7)		2.75 (1.2–7.0)	
-Abs. monos. (thousand/µL)	0.58 (0.2–0.6)		0.45 (0.4–1.3)		0.38 (0.3–0.9)		0.48 (0.3–1.2)		0.26 (0.5–1.1)	
-Abs. eos. (thousand/µL)	0.16 (0.03–0.47)		0.35 (0.0–0.4)		0.32 (0.0–0.4)		0.22 (0.0–0.3)		0.11 (0.0–0.3)	
**Immunology**										
CD19, CD20 (absolute/µL)	86		147		288		139		123	
IgD+CD27− naïve B-cells (norm ca. 75% of CD19/CD20) (% d. CD19)	45		57.14		34		70.19		51	
IgD+CD27+ unswitched memory B cells (norm >10% of CD19/CD20) (% d. CD19)	0		0		0.4		0.96		0	
IgD−CD27+ switched memory B-cells (norm ca. 10% of CD19/CD20) (% d. CD19)	23		32.5		48.82		20.2		33	
NK cells	normal		normal		normal		normal		normal	
IgG (subclasses 1–4 included), IgA, IgM	normal		normal		normal		normal		normal	
T cell subpopulation	normal		normal		normal		normal		normal	
INFg expression of stimulated memory CD4+ T cells with PMA/ionomycin	slightly decreased		normal		normal		normal		normal	
IL-4 expression of stimulated memory CD4+ T cells with PMA/ionomycin	normal		normal		normal		high-normal		high-normal	
IL-17 expression of stimulated memory CD4+ T cells with PMA/ionomycin	normal		normal		normal		normal		normal	
IL-2 expression of stimulated memory CD4+ T cells with PMA/ionomycin	normal		normal		normal		normal		normal	
CD4+ and CD8+ T cell proliferation after PHA/anti-CD3 +/− CD28 stimulation	normal		normal		normal		normal		normal	
ANA	✗		✗		✗		✗		✗	
ENA-screen, ANCAs, ds-DNA antibodies	✗		n.a.		n.a.		n.a.		✗	
Rheumatoid factor and anti-CCP antibodies	✗		n.a.		n.a.		n.a.		n.a.	
Cryoglobulins and cold aggultinins	✗		n.a.		n.a.		n.a.		n.a.	
Myositis antibodies *	✗		n.a.		n.a.		n.a.		n.a.	
**Additional Examinations**										
Complement factors (C3, C4)	✗		n.a.		n.a.		n.a.		n.a.	
TSH (mU/l; reference 0.7–4.17)	1.26		2.25		2.96		3.12		2.35	
fT3 (pg/mL; reference 2.79–4.42)	n.a.		3.9		4.0		4.4		4.4	
fT4 (ng/mL)	11.7 (9.3–17)		0.9 (0.8–1.2)		0.8 (0.8–1.2)		1.1 (0.8–1.2)		n.a.	
HIV-serology	negative		negative		negative		negative		negative	
**Vaccine Responses**										
Anti-tetanus toxin IgG (IU/mL)	1.03		0.95		0.92		insufficient (0.13)		0.42	
Anti-diphterie toxin IgG (IU/mL)	1.20		insufficient (0.22)		0.33		insufficient (<0.10)		0.41	
Anti-HBs (m/U/mL)	>1000		263		insufficient (17)		>1000		>1000	
Measles IgG	✓		✓		✓		✓		✓	
Mumps IgG	✓		✓		✓		✓		✓	
Rubella IgG Clia (IU/mL)	n.a.		129		70.3		181		>350	
VZV IgG	✓		✓		✓		✓		✓	

Abbreviations: f *female*; m *male*; ✓ *present*; ✗ *not present*; CRP *c reactive protein*; IL-2 R *interleukin-2 receptor*; CK *creatine kinase*; U *Units*; mL *milliliter*; L *liter*; mg *milligram*; dL *deciliter*; µg *microgram*; µL *microliter*; % *percentage*; Abs. *absolute*; neutros *neutrophils*; lymphos *lymphocytes*; monos *monocytes*; eos *eosinophils*; NK *cells natural killer cells*; Ig *Immunoglobulin*; PHA *phytohaemagglutinin*; PMA *phorbol 12-myristate 13-acetate*; ANA *antinuclear antibodies*; ENA *extractable nuclear antibodies*; ANCA *anti-neutrophilic cytoplasmatic antibodies*; ds-DNA-AK *double strain desoxyribonucleoid acid antibodies*; anti-CCP AK *anti-citrulline peptide antibodies*; HIV *human immunodeficiency virus*; TSH *thyroid-stimulating hormone*; fT3 *free triiodothyronine*; fT4 *free tetraiodothyronine*; HB *hepatitis* B; VZV *varicella zoster virus*; * myositis antibodies: *anti-EJ, anti Jo-1, anti-MAD5, anti-Mi-2, anti-NXPL, anti-OJ, anti-PL-12, anti-PL7, anti-Pre-100, anti-Ro-S2, anti-SAE-1, anti-PM-75, anti-SRP and anti-TIF19*.

**Table 2:** Clinical and immunological characteristics of previously described patients with *PLCG2* gene variants diagnosed as PLAID and APLAID.

Clinical and Immunological Characteristics	PLAID				APLAID												
	Ombrello et al. [3]	* Wang et al. [10]		Kutukculer et al. [18]		Zhou et al. [5]	Neves et al. [9]	Moran- Villasenor et al. [12]	Novice et al. [14]			Khabbazi et al. [15]	Martin-Nalda et al. [8]		Suri et al. [16]		Wu et al. [17]
	n = 27	n = 1	n = 1	n = 1	n = 1	n = 2	n = 1	n = 1	n = 3			n = 1	n = 1	n = 1	n = 1	n = 1	n = 1
**Clinical Symptoms**																	
Headache	n.a.	n.a.	n.a.	n.a.	n.a.	n.a.	n.a.	n.a.	n.a.	n.a.	n.a.	✓	n.a.	n.a.	n.a.	n.a.	✓
Sensorineural deafness	n.a.	✗	✗	n.a.	n.a.	n.a.	✓	n.a.	n.a.	n.a.	n.a.	n.a.	n.a.	n.a.	n.a.	n.a.	✗
Eye inflammation	n.a.	✗	✗	n.a.	n.a.	✓	✓	✓	✗	✓	✗	n.a.	✓	✓	✗	✓	✗
Cold urticaria	✓	✓	✓	n.a.	✓	✗	✗	✗	✗	n.a.	✗	✗	n.a.	✗	n.a.	n.a.	n.a.
Rashes	urticaria	urti-caria	urti-caria	n.a.	urticaria, erythem, ovaloid scars	erythmatous plaques, vesiculo-pustular lesions	vesiculo- pustular rash	vesiclo- pustular rash	erythematous, non-folliculo- centric papules, urticaria	poly- morphic eruption	✗	urticaria	erythematous plaques, vesiculo- pustular	maculo- papular eruption, erythematous plaques, urticaria	erythematous macular rash	photo- sensitive rash	vesiculopustular rashes
Cutis laxa	n.a.	n.a.	n.a.	n.a.	n.a.	n.a.	✓	n.a.	n.a.	n.a.	n.a.	n.a.	✓	✓	n.a.	n.a.	n.a.
Cutaneous granulomas	n.a.	n.a.	n.a.	n.a.	n.a.	n.a.	✓	✓	n.a.	n.a.	n.a.	n.a.	✓	n.a.	n.a.	n.a.	n.a.
Inflammatory bowel disease	n.a.	n.a.	n.a.	n.a.	n.a.	✓	✓	✓	✓	n.a.	n.a.	✗	✗	✗	n.a.	n.a.	✗
Abdominal pain	n.a.	✗	✗	n.a.	n.a.	✓	n.a.	n.a.	n.a.	n.a.	n.a.	✓	✗	✗	n.a.	n.a.	✗
Arthralgia/myalgia	n.a.	✗	✗	n.a.	n.a.	✓	n.a.	n.a.	n.a.	✓	n.a.	✓	✗	✗	✓	✓	✗
Allergic disease	✓(15/27)	n.a.	n.a.	n.a.	✗	n.a.	✗	n.a.	n.a.	n.a.	n.a.	n.a.	n.a.	n.a.	n.a.	n.a.	✗
Autoimmunity	✓	✗	✗	n.a.	✗	✗	✗	✗	✗	n.a.	n.a.	✗	✗	✗	n.a.	n.a.	✗
Recurrent (chest) infection	✓	✗	✗	✓	n.a.	✓	✓	✓	✓	✓	✓	n.a.	✓	✓	✓	n.a.	✗
Interstitial pneumonitis	n.a.	n.a.	n.a.	n.a.	n.a.	✓	✓	n.a.	✗	n.a.	✓	n.a.	n.a.	n.a.	✓	n.a.	✗
**Immunology**																	
T cells	norm	n.a.	n.a.	norm	norm	norm, INF-y/IL-17 prod. norm	norm, INF-y/IL-17 prod. ↓	norm	norm	n.a.	norm	n.a.	norm	↓	n.a.	n.a.	n.a.
Class-switched memory B cells	↓	n.a.	n.a.	n.a	n.a.	↓	↓	↓	↓	↓	↓	n.a.	n.a.	↓	n.a.	n.a.	n.a.
NK cells	↓	n.a.	n.a.	norm	n.a.	norm	norm	↓	n.a.	n.a.	norm	n.a.	norm	norm	n.a.	n.a.	↓
IgG	↓	n.a.	n.a.	norm	↑	n.a.	↓	↓	n.a.	n.a.	↓	norm	↓	norm	↑	n.a.	↓
IgA	↓	n.a.	n.a.	norm	↑	n.a.	↓	↓	n.a.	n.a.	↓	norm	↓	norm	↑	↑	↓
IgM	↓	n.a.	n.a.	↓	norm	n.a.	↓	↓	↓	↓	↓	norm	↓	↓	norm	norm	↓
Circulating auto antibodies	✓(13/27)	✓	✓	n.a.	✗	✗	✗	✗	✗	n.a.	n.a.	✗	✗	✗	✗	✗	✗

Abbreviation: * Authors address diseases as FCAS3 (in line with https://www.omim.org/entry/614468 assessed on 8 May 2022 synonym PLAID); ✓ *symptom present*; ✗ *symptom not present*; ↓ *values decreased*; ↑ *values increased*; n.a. *not available*; NK *cells natural killer cells*; IgG/IgA/IgM *immunoglobulin G/A/M*; INF *interferon*; IL-17 *interleukin-17*; prod *production.*

**Table 3:** Genetic and functional characteristics of previously described *PLCG2* gene variants causing PLAID and APLAID phenotype.

Authors	Disease	*PLCG2* Gene Variant	Pathogenicity Classification	Functional Test
Ombrello et al. [3]	PLAID	c.1935-521_2054+5385del/c.1935-322_2054+4100del, p.W646_R685del; c.2055-1396_2417+2699del, p.A686_R685.del	not classified ^1^	enzymatic activity of PLCy2 in a COS-7 transfected system, measurement of calcium flux, phosphorylation of ERK, and degranulation of natural killer cells
Wang et al. [10] *	PLAID	c.3244T>C, p.C1082R	not classified ^1,Δ^	n.a.
	PLAID	c.3524T>A, p.I1175K		
Kutukculer et al. [18]	PLAID	c.415C>T, p.P139S	VUS [18]	n.a.
	APLAID	n.a., p.R268A	benign [18]	
Zhou et al. [5]	APLAID	c.2120C>A, p.S707Y	not classified ^1^	transfection of HEK293T/ COS-7 cells, measurement of enzymatic activity of PLCy2, calcium flux, production of IP_3_/IP_1_ and ERK phosphorylation
Neves et al. [9]	APLAID	c.2543T>C, p.L848P	not classified ^1^	transfection of COS-7 cells, measurement of IP and enzymatic activity of PLCy2
Moran- Villasenor et al. [12]	APLAID	c.2543T>C, p.L848P	not classified ^1^	n.a.
Novice et al. [14]	APLAID	c.3422T>A, p.M1141K	pathogenic [14]	calcium flux, ERK phosphorylation, platelet activation related to PLCy2 and BCR stimulation
Khabbazi et al. [15]	APLAID	c.579C>G, p.H193Q	pathogenic (homzy.) [15]	n.a.
Martin-Nalda et al. [8]	APLAID	c.2533_2544del, p.L845_L848del	pathogenic ^1^	calcium flux, measurement of IP, enzymatic activity of PLC and cytokine measurements after LPS stimulation
	APLAID	c.2122G>C, p.A708P		
Suri et al. [16]	APLAID	c.2393A>G; p.N798S	not classified ^1^	n.a.
Wu et al. [17]	APLAID	c.505A>G, p.I169V	VUS ^1,□^	n.a.

Abbreviations: PLAID PLCG2-associated antibody deficiency and immune dysregulation; APLAID autoinflammation and PLCG2-associated antibody deficiency and immune dysregulation; * authors address diseases as FCAS3 (in line with https://www.omim.org/entry/614468 assessed on 8 May 2022 synonym PLAID); VUS variant of uncertain significance; homzy homozygeous; PLCG2 phospholipase Cγ2; ERK extracellular signal-regulated kinase; HEK human embryonic kidney; IP_3_ inositol-1,4,5-trisphosphate; ^1^ https://infevers.umai-montpellier.fr/web/search.php?n=14 assessed on 1 May 2022; ^Δ^ listed in Infevers as APLAID; ^□^ listed in Infevers as undefined autoinflammatory diseases phenotype.

**Table 4:** *PLCG2* variants that might be associated with some APLAID/ PLAID-like characteristics.

	Deza et al. [19]			Christiansen et al. [20]	Mahajan et al. [21]	Park et al. [11]
	n = 1	n = 1	n = 1	n = 1	n = 1	n = 1
Leading symptom/suggested disease reported	PLAID/acquired cold urticaria			idiopathic thrombocytopenia, pneumococcal meningitis	autoinflammatory epidermolysis bullosa	recurrent skin blistering disease and B-cell lymphopenia
*PLCG2* gene variant	c.3125G>C, p.S1042T	c.1274T>G, p.F425C	c.1565C>G, p.P522R	c.2866C>T, p.R956C	c.2393A>G, p.N798S	c.2119T>C, pS.707P
Pathogenecity classification	likely benign	VUS	likely benign	VUS ^1^	not classified^1^	likely pathogenic [11]/likely benign ^1^
Comment	n.a			additional Variant *TNFRSF1A,* c.1343C>T, p.P448L	n.a.	n.a.
**Clinical Symptoms**						
Headache	n.a.	n.a.	n.a.	n.a.	n.a.	n.a.
Sensorineural deafness	n.a.	n.a.	n.a.	n.a.	n.a.	n.a.
Eye inflammation	n.a.	n.a.	n.a.	n.a.	n.a.	n.a.
Cold urticaria	✓	✓	✓	n.a.	n.a.	n.a.
Rashes	urticaria	urticaria	urticaria	n.a.	n.a.	blistering skin lesions
Cutis laxa	n.a.	n.a.	n.a.	n.a.	n.a.	n.a.
Cutaneous granulomas	n.a.	n.a.	n.a.	n.a.	cutaneous erosions, depigmentation	n.a.
Inflammatory bowel disease	n.a.	n.a.	n.a.	n.a.	✗	n.a.
Abdominal pain	✓	✗	✗	n.a.	n.a.	n.a.
Arthralgia/myalgia	✗	✗	✗	n.a.	joint destruction	n.a.
Allergic disease	n.a.	n.a.	n.a.	n.a.	n.a.	n.a.
Autoimmunity	n.a.	n.a.	n.a.	n.a.	✗	n.a.
Recurrent (chest) infection	✗	✗	✗	n.a.	n.a.	✓
Interstitial pneumonitis	n.a.	n.a.	n.a.	✓	✗	n.a.
**Immunology**						
T cells	n.a.	n.a.	n.a.	norm.	n.a.	norm.
Class-switched memory B cells	n.a.	n.a.	n.a.	↓	n.a.	n.a.
NK cells	n.a.	n.a.	n.a.	norm.	n.a.	norm.
IgG	n.a.	n.a.	n.a.	↓	n.a.	norm.
IgA	n.a.	n.a.	n.a.	↓	n.a.	norm.
IgM	n.a.	n.a.	n.a.	norm.	n.a.	↓
Circulating auto antibodies	n.a.	n.a.	n.a.	n.a.	✗	✗

Abbreviations: PLAID *PLCG2-associated antibody deficiency and immune dysregulation*; APLAID *autoinflammation and PLCG2-associated antibody deficiency and immune dysregulation*; VUS *variant of uncertain significance, ✓ symptom present*; ✗ symptom not present; ↓ values decreased; n.a. *not available*; norm. *normal value*; NK cells *natural killer cells*; IgG/IgA/IgM *immunoglobulin G/A/M*; ^1^ https://infevers.umai-montpellier.fr/web/search.php?n=14 assessed on 1 May 2022.

## Data Availability

All data are incorporated in the article and its online Supplementary Material.

## Supplementary Materials

The following supporting information can be downloaded at: https://www.mdpi.com/article/10.3390/jcm11154369/s1, S1: Targeted exome sequencing, S2: Immunological testing, S3: Additional variant information, S4: Modelling of the PLCG2 protein structure.
