# Supplementary material for: Variant in the PLCG2 Gene May Cause a Phenotypic Overlap of APLAID/PLAID: Case Series and Literature Review
Source: J Clin Med. 2022 Jul 27;11(15):4369. doi: 10.3390/jcm11154369 (PMC9368933; doi:10.3390/jcm11154369)
Supplement: Supplementary file 1 [file jcm-11-04369-s001.zip › jcm-1833379-Supplementary.pdf]

Supplementary material

S1: Targeted exome sequencing.

Exome sequencing and variant filtering:

Exome sequencing was performed on genomic DNA from the four affected siblings. Coding regions were enriched using SureSelect XT Human All Exon V7 kits (Agilent Technologies, Santa Clara, CA, USA) and prepared libraries were sequenced as 2x100 bp paired-end reads on a NovaSeq 6000 system (Illumina, San Diego, CA, USA) to a 93.95 to 120.06 X coverage of the target region. Additional quality metrics on the performed experiments are shown in Table 1.

Generated sequences were analyzed using the megSAP pipeline (https:// github. com/ imgag/ megSAP). Different filtering steps were performed to prioritize putatively clinically relevant DNA variants including a search for rare (MAF < 0.01 % in gnomAD (https://gnomad.broadinstitute.org; assessed January 2022) and an in-house database) heterozygous non-synonymous changes shared by all affected individuals. This approach left a total of 17 heterozygous variants in 16 genes. Of these, 6 have been previously associated with human diseases, namely *PTCH2, POMGNT1, EPRS1, TREX1, PLCG2,* and *HCLS*. Of these, *PLCG2* was the only gene that has been previously reported in the context of the clinical features observed in the investigated family with an autosomal dominant mode of inheritance. Datasets were analyzed by two trained diagnostic molecular geneticists, revealing no other likely disease-causing variants.

Table S1: Exome sequencing quality metrics of patients 2 – 5.

parameter	value (patient 2)	value (patient 3)	value (patient 4)	value (patient 5)
Q20 read percentage	99.97	99.97	99.97	99.98
gc content percentage	50.44	50.67	50.98	50.89
variant count	43226	43397	43326	44273
known variants percentage	99.66	99.66	99.66	99.65
transition/transversion ratio	2.61	2.63	2.63	2.61
mapped read percentage	99.16	99.49	99.63	99.63
on-target read percentage	68.62	69.38	69.99	69.27
properly-paired read percentage	97.97	98.44	98.78	98.74
insert size	197.77	199.09	199.41	198.34
target region read depth	98.73	110.26	93.95	120.06
target region 20x percentage	94.39	95.02	93.97	95.55
bases sequenced (MB)	11992.05	13600.44	11277.94	14539.99

Antibody	Clone	Fluorochromes	Company
anti-human CD27	L128	Allophycocyanin (APC)	BD Biosciences
anti-human CD38	HB7	Fluorescein isothiocyanate (FITC)	BD Biosciences
anti-human CD19	4G7	PerCP	BD Biosciences
anti-human IgD	IA6-2	Phycoerythrin (PE)	BD Biosciences

S2: Immunological testing.

Cytokine staining:

To measure cytokine production, PBMCs (peripheral blood mononuclear cells) were stimulated with phorbol 12-myristate 13-acetate (PMA, 50 ng/ml, Merck) and ionomycin (1 µg/ml, Merck) in the presence of Brefeldin A (GolgiPlug, BD Biosciences) for 4 h at 37°C. Then cells were harvested and stained with antibodies against CD45RO, CD3 and CD4 for 15 min at room temperature. Subsequently, the Cytofix/Cytoperm™ Plus Fixation/Permeabilization Kit (BD Biosciences) was used for intracellular staining of IL-2, IL-4, IL-17 and IFN-γ. Cells were acquired on a Navios flow cytometer (Beckman Coulter).

Antibody	Clone	Fluorochromes	Company
anti-human CD3	SK7	PerCP	BD Biosciences
anti-human CD45RO	UCHL1	PE-CF594	BD Biosciences
anti-human CD4	SFCI12T4D11	PC7	Beckman Coulter
anti-human IL-4	8D4-8	APC	eBioscience
anti-human IL-17	eBio64DEC17	PE	eBioscience
anti-human IFN-γ	B27	FITC	BD Biosciences
anti-human IL-2	5344.111	PE	BD Biosciences

T-cell proliferation:

For analysis of T-cell proliferation, PBMCs were labeled with CFSE (Molecular Probes) for 15 min at 37°C. Cells were seeded into a 96-well flat bottom plate and left untreated or stimulated with PHA (2.5 µg/ml, Remel), 300 ng/ml plate bound anti-CD3 antibody (Clone OKT3, eBiosciences), 1000 ng/ml plate bound anti-CD3 antibody or 300 ng/ml plate bound anti-CD3 antibody and 1 µg/ml soluble anti-CD28 antibody (Clone CD28.2, BD Biosciences). Cells were harvested after 4-6 days, stained with antibodies against CD8 and CD4 and acquired on a Navios flow cytometer (Beckman Coulter).

Antibody	Clone	Fluorochromes	Company
anti-human CD4	SFCI12T4D11	PC7	Beckman Coulter
anti-human CD8	RPA-T8	APC	BD Biosciences

S3: Additional variant information.

Gene	Transcript-ID	Variant	Status	gnomAD frequency	Varsome classification	ClinVar interpretation
PLCG2	ENST00000359376	c.77>T, p.Thr26Met	heterozygote	0.0006	benign	Conflicting (VUS, 2x, benign1x, likely benign 1x)

Abbreviation: PLCG2 *Phospholipase-Gamma-2,* VUS *variant of uncertain significance*

S4: Modelling of the PLCG2 protein structure.

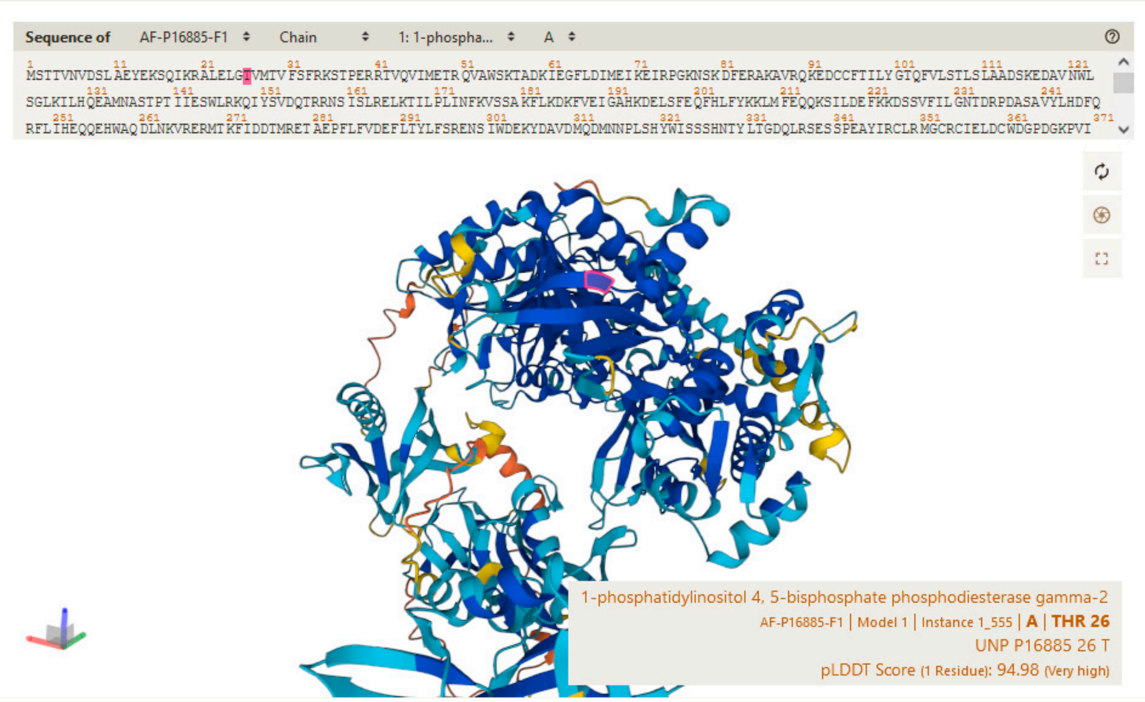

source: https://alphafold.ebi.ac.uk/entry/P16885

AlphaFold predicts for the missense changes a location within a beta-sheet structure with very high confidence (pLDDT > 90). The aliphatic amino acid Threonin is replaced by the amino acid Methionin, potentially disrupting the secondary structure. However, the amino acid changes are not predicted to be highly damaging (CADD-score = 21, CADD version 1.5).
